# Laparoscopic ovarian electrocautery versus gonadotropin therapy in infertile women with clomiphene citrate-resistant polycystic ovary syndrome: A systematic review and meta-analysis

**Published:** 2014-08

**Authors:** Zahra Moazami Goudarzi, Hossein Fallahzadeh, Abbas Aflatoonian, Masoud Mirzaei

**Affiliations:** 1 *International Campus, Shahid Sadoughi University of Medical Sciences, Yazd, Iran.*; 2 *Department of Biostatistics and Epidemiology, Research Center of Prevention and Epidemiology of Non-Communicable Disease, Faculty of Health, Shahid Sadoughi University of Medical Sciences, Yazd, Iran.*; 3 *Research and Clinical Center for Infertility, Shahid Sadoughi University of Medical Sciences, Yazd, Iran.*; 4 *Yazd Cardiovascular Research Centre, Shahid Sadoughi University of Medical Sciences, Yazd, Iran.*

**Keywords:** *Ovarian drilling*, *Polycystic ovary syndrome*, *Meta-analysis*, *Gonadotropin*, *Infertility*, *Clomiphenecitrate*

## Abstract

**Background:** Some trials have compared laparoscopic ovarian drilling (LOD) with gonadotropins but, because of variations in study design and small sample size, the results are inconsistent and definitive conclusions about the relative efficacy of LOD and gonadotropins cannot be extracted from the individual studies.

**Objective:** To evaluate the relative efficacy of LOD and gonadotropins for infertile women with clomiphene citrate- resistant poly cystic ovary syndrome (PCOS).

**Materials and Methods: **A complete electronic literature search in databases including EMBASE, MEDLINE, Cochrane Library and Google scholar for some specific keywords was accomplished. We contained randomized clinical trials comparing outcomes between LOD, without medical ovulation induction, and gonadotropins.

**Results: **Six trials, covering 499 women, reported on the primary outcome of pregnancy rate. There was no evidence of a difference in pregnancy rate when LOD compared with gonadotropins (OR: 0.534; 95% CI: 0.242-1.176, p=0.119, 6 trials, 499 women, I^2^=73.201%). There was evidence of significantly fewer live births following LOD compared with gonadotropin (OR: 0.446; 95% CI: 0.269-0.74, p=0.02, 3 trials, 318 women, I^2^=3.353%). The rate of multiple pregnancies was significantly lower in the LOD arm compared to the gonadotropins arm (OR: 0.127; 95% CI: 0.028-0.579, p=0.008, 3 trials, 307 women, I^2^=0%).

**Conclusion:** Our result revealed that there was no evidence of a significant difference in rates of clinical pregnancy and miscarriage in women with clomiphene citrate-resistant PCOS undergoing LOD compared to the gonadotropin arm. The decrease in multiple pregnancies rate in women undergoing LOD makes this option attractive. The increase in live birth rate in the gonadotropin group may be because of the higher rate of multiple pregnancies in these women. However, more focus on the long-term effects of LOD on ovarian function is suggested.

## Introduction

Infertility is defined as one year unprotected intercourse without pregnancy. This problem affects 10-15% of young couples around the world (1, 2). Female factors were more common (57.5%) in etiologic assessment of infertility (3). Ovulatory dysfunction is a common cause of infertility and poly cystic ovary syndrome (PCOS) is the main reason of this dysfunction. PCOS is the most common endocrinopathy in women of childbearing age. The prevalence of this syndrome is approximately 10% of women in their reproductive years. Clinical signs of PCOS are menstrual disorder, oligo-ovulation or anovulation, hirsutism, acne, and in severe cases alopecia. Obesity is common in this syndrome but it is not universal. Although the primary cause in PCOS has remained unclear, a genetic factor is suspected to play a role in the etiology of the disease (4, 5). Therapeutic approaches to chronic anovulation from PCOS are still under debate. The drug of first choice for ovulation inclusion is clomiphene citrate (CC), taken orally. However, about 20% of women are failed to ovulate in spite of increasing doses of this drug, up to 150 mg daily (6, 7). 

Ovulation induction with gonadotropins and laparoscopic ovarian electro cautery laparoscopic ovarian drilling (LOD) are the alternative treatments for women with CC-resistant PCOS. But both of these protocols have some disadvantages. Gonadotropins increase the risk of serious side effects, such as multiple pregnancies and the ovarian hyperstimulation syndrome (OHSS). Although these risks are avoided with LOD but the need for a surgical procedure and the possible long term effect on ovarian function and creation of tubo-ovarian adhesions are some of disadvantages of this surgical treatment (-).

Some trials have compared LOD with gonadotropins but, because of variations in design study, the results were inconsistent. Also, because of small sample size, definitive conclusions about the relative efficacy of LOD and gonadotropins cannot be extracted from the individual studies (-). The aim of this systematic review and meta-analysis was to compare the effectiveness and safety of LOD with gonadotropins when used for infertile women with CC-resistant PCOS.

## Materials and methods

To report the results of this meta-analysis, the Preferred Outcome Items for Systematic Review and Meta-analysis (PRISMA statement) was used (17).

**Search Strategy**

Publications described randomized controlled trials of LOD versus gonadotropins for infertile women with CC-resistant PCOS were sought. This was done by a complete electronic literature search without time limitation in EMBASE, MEDLINE, Cochrane Library and Google scholar. Also the main ongoing clinical trials registries, such as: clinicaltrials.gov, irct.ir, controlled-trial.com and trialscentral.org were probed. Then bibliographies of relevant publications for finding more articles were considered (18). The keywords were polycystic ovary syndrome, laparoscopic ovarian drilling, infertility and gonadotropin.

**Selection Criteria and Data Extraction**

We included randomized controlled trials of infertile women with CC-resistant PCOS who undertook LOD or gonadotropin in order to induce ovulation. Methodological quality of all trials was evaluated by the standard scoring system developed by Jadad and colleagues (19). The screening of studies, selection, data extraction, validation and the assessment of methodological quality were performed independently by two authors (Z.M.G., H.F). Disagreements in the assessment of individual trials were largely due to reading errors and were solved through discussion.

**Outcome Measure**

The primary end point was pregnancy rate per women randomized, identified by serum β-HCG of >50 IU/L and gestational sac seen on ultrasound. Secondary outcomes were live birth rate, multiple pregnancies rate and miscarriage rate per women randomized.

**Statistical analysis**

The effectiveness of LOD compared with using gonadotropin was measured as an odds ratio (OR) for each outcome, with corresponding 95% confidence interval (CIs). Statistical significant was set at a p<0.05. Heterogeneity of treatment effect was tested with I^2^ index (20). The fixed-effects model was used, unless significant heterogeneity was present, in which case the random-effects model was used to pool the data. Funnel plot analysis was applied to detect publication bias. Statistical analysis was performed using Review Manager (Ver. 5.2) software provided by the Cochrane Collaboration for dichotomous data (21).

## Results

**Figure 1:**
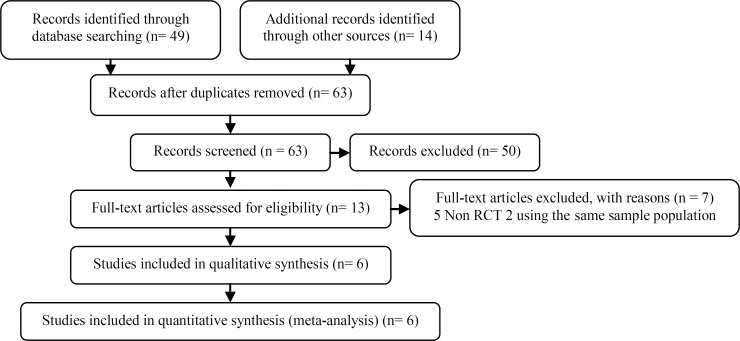
PRISMA flow diagram detailing selection of studies for inclusion. RCT refers to randomized controlled trial

The electronic search identified 49 relevant articles and 14 additional papers identified through other sources. After screening of title and abstract, 50 of them were excluded for a variety of reasons such as, duplication, lack of randomization, unavailable data and concomitant use of clomiphene citrate or rFSH in LOD group, if ovulation was failed. In two studies all results and data with details were reported for each sub-group, including: LOD and LOD with other treatment. Based on the agreement of the authors, their data were used (Z.M.G., A.A.) (11, 15). After reviewing the full texts of the remaining articles, five were excluded because there were not RCTs and two trials with the same raw data were exclude (7, 16, -). Therefore, six trials were eligible for inclusion in the meta-analysis (-). The complete selection progress is displayed in Figure 1.

**Description of Included studies**

Six trials met the inclusion criteria; all of them had more than 30 participants. The important prognostic factor of age was reported in all published trials and was similar across all the included trials. Mean of body mass index (BMI) was reported in all and ranged from 26-29.5 Kg/m^2^ (-). All trials assessed pregnancy rate, in three trials with 318 women live birth rate were reported too. Multiple pregnancy rates were estimated in three trials covering 307 women and the rate of miscarriage was calculated in four trials with 422 samples. Further information on the trials included in this meta-analysis is given (Table I). 

**Study Quality and Publication Bias**

Quality of all trials was rated by Jadad score. This scale rates studies on the basis of the quality of randomization, double-blinding and inclusion of data on dropouts and withdrawals. The range of the scale is 0-5, with 0 denoting poor quality and 5 denoting the highest quality, the cut off point for this score is 2 (19). Because blinding of treatment was not possible, maximum score in this review was 3 (Table II). Two trials provided intention-to-treat analyses (11, 12). The funnel plot demonstrated a symmetrical inverse funnel shape, indicating that selective bias is unlikely in this meta-analysis.

**Effects of interventions**

**Figure 2:**
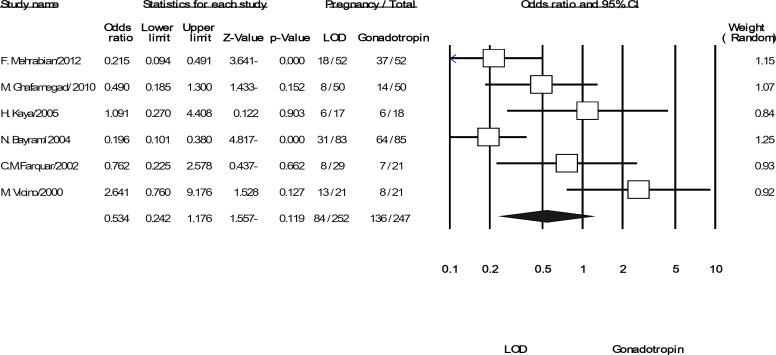
The confidence interval (95% confidence interval, 0.24–1.17) and odds ratio (OR: 0.53) of the pregnancies per women randomized in each of the studies and. Heterogeneity I^2^ =73.2% (df=5), p=0.002

**Figure 3:**
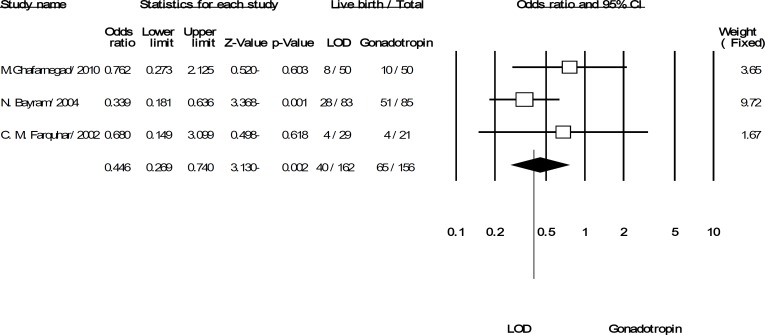
The confidence interval (95% confidence interval, 0.26–0.74) and odds ratio (OR: 0.44) of the live birth per women randomized in each of the studies and. Heterogeneity I2 =3.35% (df=2), p=0.35

Overall, six trials covering 499 women, included in the pooled analysis for the discussed outcomes. All trials reported the primary outcome of pregnancy rate per women randomized. Although pregnancies were reported in 33% of women in the LOD arm and 55% in gonadotropins arm but overall summery effect was OR: 0.534 (95% CI: 0.242-1.176, p=0.119, 6 trials, 499 women, I^2^=73.201%). The result indicated that there was no evidence of significant difference in pregnancies when LOD compared with gonadotropins (Figure 2). This pattern of result was changed for the rate of live birth, OR: 0.446 (95% CI: 0.269-0.74, p=0.002, 3 trials, 318 women, I^2^=3.353%). It showed that, LOD was resulted a statistically significant decrease in the live birth rate compared to gonadotropins (Figure 3). 

**Figure 4:**
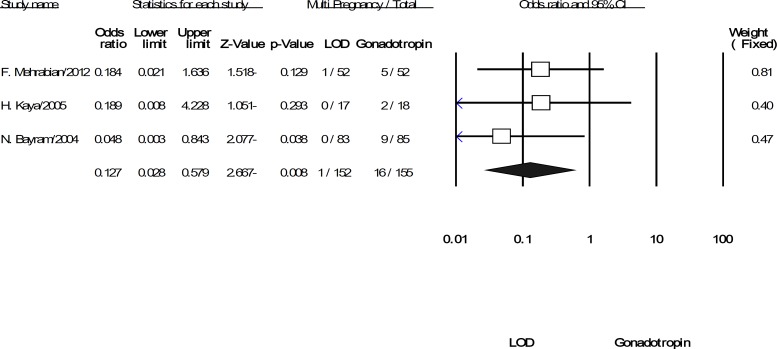
The confidence interval (95% confidence interval, 0.028–0.57) and odds ratio (OR: 0.12) of the multiple pregnancies per women randomized in each of the studies and. Heterogeneity I^2^ =0% (df=2), p=0.73

**Figure 5:**
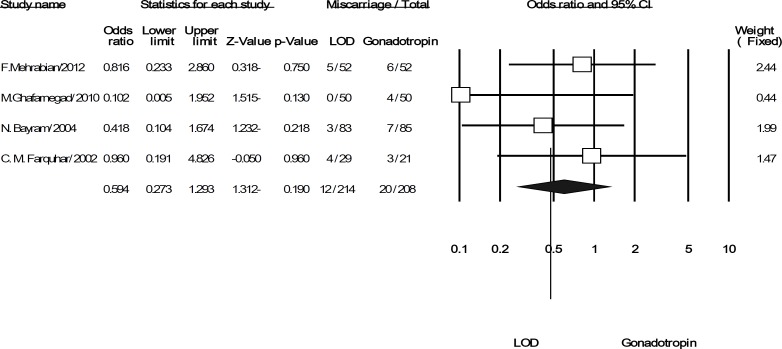
The confidence interval (95% confidence interval, 0.27–1.29) and odds ratio (OR: 0.59) of the miscariage per women randomized in each of the studies and. Heterogeneity I2 =0% (df=2), p=0.53

Pooling the data from the three included trials was showed significant difference in multi pregnancies for LOD compared to gonadotropins (OR: 0.127; 95% CI: 0.028-0.579, p=0.008, 3 trials, 307 women, I^2^=0%). It means that LOD can reduce the odds of multiple pregnancies versus using gonadotropin (Figure 4). Finally, the difference rate of miscarriage between the LOD groups compared to using gonadotropins was not statistically significant (OR: 0.594; 95% CI: 0.273-1.293, p=0.19, 4 trials, 422 women, I^2^=0%) (Figure 5).

**Table I:** Characteristics of the clinical trial included in the review (LOD/Gonadotropins)

**Author/Year**	**Patient (N)**	**Age (year)**	**BMI (kg/m** ^2^ **)**	**Duration of trial (month)**	**Outcomes**
F. Mehrabian/ 2012 (16)	104 (52/52)	29.17 ± 5.47 28.51 ± 5.51	27.73 ± 6.16 27.55 ± 6.07	3/3	- Pregnancy - Multiple pregnancies - Miscarriage
M. Ghafarnegad/ 2010 (14)	100 (50/50)	26.8 ± 3.3 26 ± 3.5	28.4 ± 4.2 28.1 ± 6	4/4	- Pregnancy - Live birth - Miscarriage
H. Kaya/ 2005 (15)	35 (17/18)	26.3 ± 4.3 25.6 ± 4.08	27.4 ± 4.2 26.3 ± 3.2	6/6	- Pregnancy - Multiple pregnancies
N. Bayram/ 2004 (12)	168 (83/85)	28.5 ± 3.7 28.7 ± 4.1	27.9 ± 6.3 27.3 ± 8.8	12/12	- Pregnancy - Live birth - Multiple pregnancies - Miscarriage
C.M.Farquhar/ 2002 (13)	50 (29/21)	29.6 ± 4.7 29.6 ± 4.2	28.3 ± 3.9 27.8 ± 4.8	6/3	- Pregnancy - Live birth - Multiple pregnancies - Miscarriage
M. Vicino/ 2000 (17)	42 (21/21)	29.8 ± 3.2 28.1 ± 3.4	30.3 ± 3.1 30.4 ± 3.9	12/12	- Pregnancy

**Table II:** Quality assessment of included trials

**Author/Year**	**Randomization**	**Description of randomization**	**Masking**	**Description of masking**	**Description of dropouts**	**Total**
F. Mehrabian (2012)	1	1	0	0	0	2
M.Ghafarnegad (2010)	1	1	0	0	0	2
H. Kaya (2005)	1	1	0	0	1	3
N. Bayram (2004)	1	1	0	0	1	3
CM. Farquhar (2002)	1	1	0	0	1	3
M. Vicino (2000)	1	1	0	0	0	2

* According to Jadad score

## Discussion

In this systematic review, studies that compared LOD with gonadotropins treatment in infertile women with CC resistant PCOS were systematically reviewed. Pregnancy rates per women randomized favored in all trials. The overall conclusion from this meta-analysis declared that evidence was insufficient of a significant difference between LOD compared with gonadotropins in the terms of pregnancy and miscarriage. While the multiple pregnancy rates appeared to be significantly reduced following treatment with LOD like live birth rate.

A meta-analysis compared LOD with ovulation induction by other treatment strategies in patients with clomiphene citrate-resistant poly cystic ovary syndrome, using gonadotropin as one of the treatments. In the study the effect of additional treatment for LOD group was not separated and found no difference in rates for pregnancy and live birth (27). The strength of our design is that studies which survey the effectiveness of ovarian drilling without extra medical treatment and additional intervention versus gonadotropins were used. As there was no evidence of a difference for pregnancy rate for both treatment options, LOD strategy may be the treatment of choice since it reduces the risk of multiple pregnancies.

Although the rate of live birth was lower after LOD than gonadotropins, this difference was wiped if trials follow their samples for long time. This idea was supported with findings of Nahuis *et al* survey (25). They continued longitudinal follow-up for 8-12 years for 95% of original samples. At this extension follow-up, they reported live birth in 86% of women who had been allocated to LOD group versus 81% in women who had been allocated to gonadotropin (rFSH) group (p=0.63). However, LOD significantly eliminated the need for re-ovarian stimulation to have a live birth outcome (53% after LOD vs. 76% after rFSH). At the end of follow-up there had been 64 live birth in the LOD group without further stimulation, and 40 live birth in rFSH group without additional treatment (p=0.09). These results confirmed the consequences that reported from other longitudinal study (28).

Repeated spontaneous ovulation and further pregnancies, was an additional benefit of surgical method (12, 29). Also, no significant differences were observed in the rate of miscarriage indicating that LOD treatment does not place women at higher risk for this adverse event compared to gonadotropins. Our findings are comparable with some other studies reported financial assessment of these treatments. The economic evaluation studies which has done with Van Wely *et al,* Farquhar *et al* and Ghafarnegad *et al* shown that ovarian drilling was considerably less expensive than ovulation induction with taking gonadotropins (13, 29, 30). Also, there is some evidence that LOD is associated with decreased OHSS (11, 12, 14).

Furthermore, the need for continuous monitoring in ovarian induction with gonadotropins also makes surgery a preferable option, albeit the surgically-related complications associated with ovarian drilling such as adhesion formation and destruction of some ovarian tissue cannot be ignored. Meta-analysis in general has several drawbacks, such as dependence on the quality of primary analysis and dependence on sufficient numbers of eligible studies to justify statistical analysis. But with using Jadad score and searching literature with two authors separately, we tried to decrease these disadvantages. Since all studies in this meta-analysis have evaluated the short-term effects of two methods, more studies on the long-term effects of LOD on ovarian function should be done.

## Conflict of interest

For all authors, this study is just motivate as the desire for professional advancement and the wish to help patients to get the best treatment and it has not any financial gain for authors.

## Footnote

***This article extracted from M.Sc. thesis. (Zahra Moazami Goudarzi)***
